# Dr. John G. Merrow

**Published:** 1916-10

**Authors:** 


**Dr. John G. Merrow** (not listed in State nor Polk Directory) died in Utica, Aug. 28.

